# Full-colour nanoprint-hologram synchronous metasurface with arbitrary hue-saturation-brightness control

**DOI:** 10.1038/s41377-019-0206-2

**Published:** 2019-10-23

**Authors:** Yanjun Bao, Ying Yu, Haofei Xu, Chao Guo, Juntao Li, Shang Sun, Zhang-Kai Zhou, Cheng-Wei Qiu, Xue-Hua Wang

**Affiliations:** 1 State Key Laboratory of Optoelectronic Materials and Technologies, School of Physics, Sun Yat-sen University, 510275 Guangzhou, China; 2 Department of Electrical and Computer Engineering, National University of Singapore, 4 Engineering Drive 3, Singapore, 117583 Singapore; 3 NUS Suzhou Research Institute (NUSRI), Suzhou Industrial Park, 215123 Suzhou, China

**Keywords:** Metamaterials, Sub-wavelength optics

## Abstract

The colour gamut, a two-dimensional (2D) colour space primarily comprising hue and saturation (HS), lays the most important foundation for the colour display and printing industries. Recently, the metasurface has been considered a promising paradigm for nanoprinting and holographic imaging, demonstrating a subwavelength image resolution, a flat profile, high durability, and multi-functionalities. Much effort has been devoted to broaden the 2D HS plane, also known as the CIE map. However, the brightness (B), as the carrier of chiaroscuro information, has long been neglected in metasurface-based nanoprinting or holograms due to the challenge in realising arbitrary and simultaneous control of full-colour HSB tuning in a passive device. Here, we report a dielectric metasurface made of crystal silicon nanoblocks, which achieves not only tailorable coverage of the primary colours red, green and blue (RGB) but also intensity control of the individual colours. The colour gamut is hence extruded from the 2D CIE to a complete 3D HSB space. Moreover, thanks to the independent control of the RGB intensity and phase, we further show that a single-layer silicon metasurface could simultaneously exhibit arbitrary HSB colour nanoprinting and a full-colour hologram image. Our findings open up possibilities for high-resolution and high-fidelity optical security devices as well as advanced cryptographic approaches.

## Synchronous Nanoprinting-Hologram Metasurfaces: Towards full hue-saturation-brightness control

Structural colours with hue-saturation-brightness (HSB) control and independent integration with full-colour hologram open up possibilities for optical security devices and advanced cryptographic approaches. Researchers from China and Singapore propose a crystalline-silicon nanoblock metasurface that is able to control the intensity and phase of transmitted red, green, blue (RGB) lights independently. By mixing the three RGB nanoblocks with different intensity proportions together, it is possible to control the HSB value of the transmitted light, pushing the metasurface structural colour from conventional two-dimensional hue-saturation space to a real three-dimensional HSB space. Furthermore, arbitrary HSB colour printing and full-colour hologram images can be integrated in one single metasurface structure. That makes a drastic leap for singlet multifunctional metasurfaces, instead of cascading multiple metasurfaces with one for colourprinting and the other for hologram respectively.

## Introduction

Colour is one of the most important properties of human visual perception. To make a material coloured, one usually uses dye or pigment, the colour of which originates from the material selective absorption in the visible band. Another way to make colour is to use nanostructures that can constructively interfere with incident light. This phenomenon is called structural colour^1^. Various plasmonic and dielectric nanostructures have been proposed to realise structural colours^2–15^, employed in vivid colour printing images with the advantages of lower cost, reduced number of needed materials, subwavelength resolution, higher resistance to heat and radiation, etc.

**Fig. 1:**
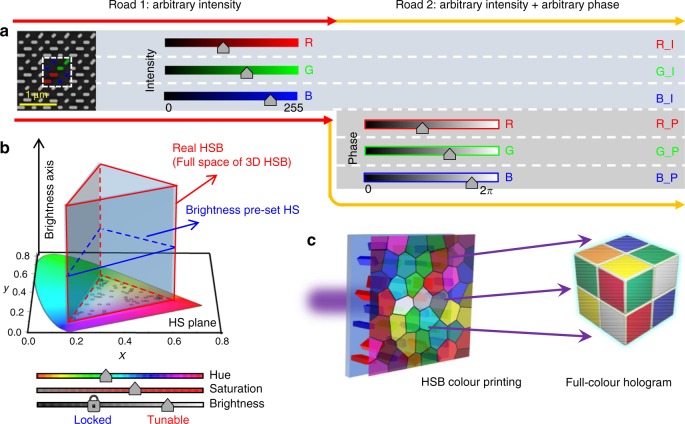
Illustration of the designed metasurface for sub-micron resolution HSB colour printing and full-colour hologram integration. **a** Scanning electron microscopy (SEM) image of a partial region of the fabricated c-silicon metasurface with the decoupled continuous intensities (road 1) and phases (road 2) of the three primary RGB components. Each pixel consists of four sub-pixels: two for blue, one for green and one for red components. **b** The full HSB colour space enabled by continuous intensity tuning of the three primary RGB components. Our method, which covers the whole RGB intensity space, equals the 3D random walk in the entire HSB space (triangular prism with red line boundaries), which is constructed from the hue and saturation (HS) plane (i.e., CIE 1931 colour space) together with a brightness axis. In comparison, the previous work lacks brightness tuning, which means that their colours can basically assemble only in a brightness pre-set HS plane (triangle with blue line boundaries). **c** HSB colour printing and full-colour hologram integration enabled by the decoupling of the intensities and phases of the three primary RGB components

An essential and instinctual developing goal is to make structural colours that are able to reproduce all the colours in the practical world. Since a colour is defined by its three inherent merits, which are the hue, saturation and brightness (HSB)^16^, a basic indispensable requirement for a structural colour technique is the on-demand generation of colours in the whole three-dimensional (3D) HSB space, which is constructed from the hue and saturation (HS) plane (i.e., CIE 1931 colour space) together with a brightness axis (shown in Fig. 1). However, in the previously demonstrated structural colours using nanostructures, real HSB colour tuning has not yet been well addressed. Specifically, in most previous works, numerous efforts have been devoted to generate various colours covering the whole visible region by changing the geometry dimensions, such as the diameter, height, and period^4–13^, which brings about only the gradual enlarging of the colour gamut in the HS plane but lacks brightness (*B*) tuning. In these structures, once the *H* and *S* values of a colour are set, the *B* value is set correspondingly, meaning their colours can basically assemble only in a certain HS plane but not in the whole 3D HSB space. Approaches based on using large-scale pixels have been proposed to address the brightness control of structural colours^14,15^; however, the final pixel sizes in these approaches are tens and hundreds of micrometres (even larger than in commercial displays), lacking the basic advantage of high resolution of the artificial structural colours. In addition, increasing the functionality of nanoprinting image technology is of great importance. To the best of our knowledge, the integration of HSB colour printing and full-colour holograms has never been reported.

Metasurfaces are two-dimensional nanostructures with the exceptional ability to control light at the nanoscale^17–28^. In this paper, we propose using crystal silicon (c-silicon) metasurfaces to realise sub-micron-resolution colour printing with HSB colour tuning (i.e., HSB colour printing) by continuous intensity tuning of the three primary colours red, green and blue (RGB). A resolution of ~36,000 dpi can be achieved in our HSB colour printing. Furthermore, in addition to the intensity control, our design also enables independent phase control of each RGB component. Thanks to this property, two arbitrary images of HSB colour printing and a full-colour hologram are integrated into the same metasurfaces. These exciting findings are believed to facilitate the development of modern image techniques with fascinating display applications.

## Results

### General concept of our HSB structural colour design

Our structural colours are formed by metasurfaces of c-silicon nanoblocks, the pixels of which consist of the three primary RGB components with continuous intensity tuning of each (Road 1 in Fig. 1a). According to the standard additive colour mixing concept with the RGB components, colour that can cover the whole RGB intensity space equals that covering the entire HSB space (Fig. 1b). In contrast, if the control over the colour intensity is neglected (which is the case in most previous studies), the brightness of a colour will be set after the design of the hue and saturation, leading to the colour tuning being limited in one brightness pre-set plane of the HSB space (2D colour tuning, Fig. 1b).

The designed metasurfaces not only enable a colour to be located in an arbitrary part of the RGB intensity space (i.e., the HSB space) but also allow the independent generation of arbitrary values in the RGB phase space (Road 2 in Fig. 1a). This fact indicates that we expand the manipulation space of the primary colours from one (intensity space) to two (intensity and phase spaces). Since phase control of the primary colours can lead to on-demand hologram images, an HSB colour printing image can be integrated with a full-colour hologram (Fig. 1c). This finding is not only a double-screen display technique but can also bring about useful applications, such as nano-steganography, with the printed image as the cover information and the hologram as the hidden information.

### Design of the three primary RGB colours

**Fig. 2:**
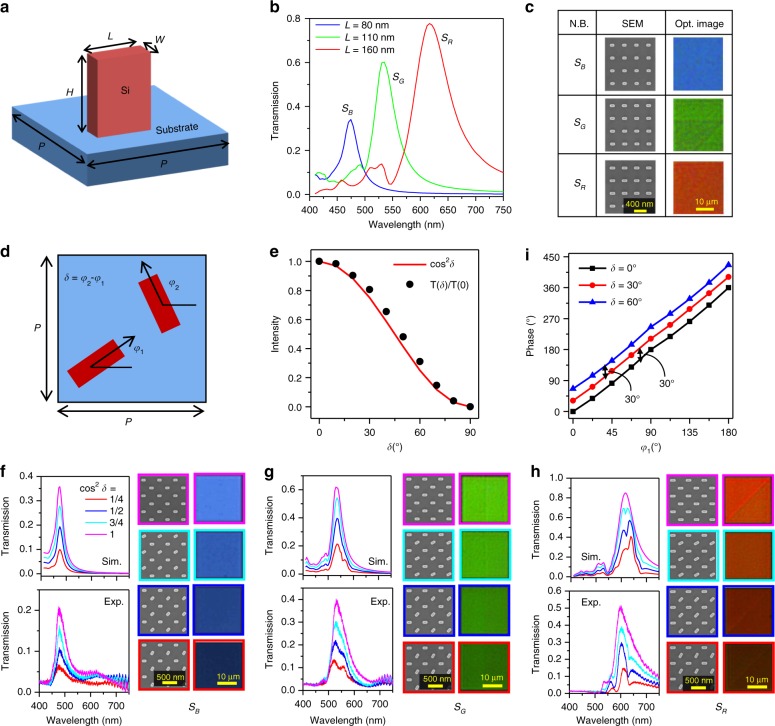
Design of c-silicon metasurface for the three primary RGB colours and continuous intensity and phase tuning. **a** Schematic of a unit cell of the silicon metasurface for the generation of the three primary RGB colours. A c-silicon nanoblock with width *W*, length *L* and height *H* stands on a fused SiO_2_ substrate. The period *P* = 400 nm. **b** The simulated cross-polarised transmission spectra of the c-silicon metasurface with *L* = 80, 110 and 160 nm. The width and height are fixed at *W* = 40 nm and *H* = 600 nm. The three types of nanoblocks are labelled *S*_B_, *S*_G_ and *S*_R_, respectively. **c** SEM and measured optical images of the fabricated c-silicon metasurface with nanoblocks *S*_B_, *S*_G_ and *S*_R_. **d** Schematic of a double-nanoblock unit cell with intensity and phase tuning for transmitted light. Each unit cell consists of two identical c-silicon nanoblocks with rotation angles of *φ*_1_ and *φ*_2_ relative to the *x-*axis. The rotation-angle difference between the two nanoblocks is defined as *δ* = *φ*_2_ − *φ*_1_. The coordinates of the centre of the left-bottom and right-top nanoblocks are (−*P*/4, −*P*/4) and (*P*/4, *P*/4), respectively. The period *P* = 550 nm. **e** The simulated intensity of zero-order transmission of the DNC with nanoblock *S*_G_ vs. *δ* with fixed *φ*_1_ = 0° (black solid circles). The wavelength chosen is the resonance wavelength of nanoblock *S*_G_. The red line shows the function of cos^2^ *δ* vs. *δ*. **f**–**h** The simulated and measured zero-order cross-polarised transmission spectra (left column), SEM figure (middle column) and measured optical images (right column) of the c-silicon metasurface with different *δ* values of DNCs with nanoblocks *S*_B_ **f**, *S*_G_ **g** and *S*_R_ **h**. **i** The simulated phase of zero-order transmitted light vs. *φ*_1_ for different *δ* values of the DNC with nanoblock *S*_G._ The phase is proportional to 2*φ*_1_

The HSB printing colour tuning is made by colour mixing the primary RGB components, which involves three steps: (1) producing three primary RGB colours, (2) continuous intensity tuning of each RGB component and (3) mixing the three RGB colours with proper intensity proportions. We first start with the generation of the three primary RGB colours. A periodical metasurface structure with a c-silicon nanoblock unit cell is designed on a fused SiO_2_ substrate (Fig. 2a). The c-silicon nanoblock has a height *H*, width *W* and length *L*. A left circularly polarised (LCP) beam is illuminated normally from the substrate side and passes through the c-silicon metasurface. The simulated transmission magnitudes of cross-polarisation (right circular polarisation, RCP) for three different nanoblocks of *L* = 80, 110 and 160 nm are shown in Fig. 2b. The details of the numerical simulations can be found in the “Materials and methods” section. As the figure shows, a single prominent resonance peak can be observed for each curve, located at approximately 470, 530 and 630. In Supplementary Fig. S1, we show the calculated colours and the corresponding positions in the CIE 1931 colour space of the three nanoblocks based on their transmission spectra^29^. The three c-silicon nanoblocks exhibit structural colours blue, green and red, which are close to the outer curved boundary in the CIE 1931 colour space, demonstrating the suitability for displaying the three primary RGB colours. We refer to the three nanoblocks as *S*_B_, *S*_G_ and *S*_R_. The obtained relative pure colour benefits from the narrow resonance peak due to the high aspect ratio (~15) of the nanoblock. If the aspect ratio decreases (*H* decreases or *W* increases), the resonance peaks may decrease, become broadband or split, resulting in a less pure colour (Supplementary Fig. S2). In comparison, the previously designed Si metasurface usually has a broadband response or has multiple resonances^9,30^, which is not suitable for the RGB primary colours. We also tested other dielectric materials, such as gallium nitride (GaN) and titanium dioxide (TiO_2_), and no optimal geometries with a single narrow resonance peak were found (Supplementary Figs. S3 and S4).

For the three different nanoblocks, the magnitude of the transmission peak decreases (Fig. 2b) as the resonance wavelength becomes shorter due to the increased absorption of silicon. The peak value of the nanoblock *S*_B_ decreases down to a magnitude of 0.35. However, this transmission value is significantly larger compared with that of amorphous silicon (a-silicon) material, which has a much larger loss than does c-silicon. If replaced with a-silicon, the transmission of nanoblock *S*_b_ is nearly zero in the entire visible frequency range (Supplementary Fig. S5); therefore, blue cannot be produced. For example, a metasurface designed with a-silicon elements^31^ can be resonant only in the green and red regions of the spectrum. As a consequence, c-silicon must be used for the metasurface design to achieve the three primary RGB colours.

To fabricate the c-silicon metasurface, we transfer the c-silicon from a silicon-on-insulator (SOI) wafer to a fused SiO_2_ substrate by the adhesive wafer bonding and deep reactive ion etching (DRIE) method^32,33^, which was used in our previous work^34^. Then, electron beam lithography (EBL) is used to define the pattern. The details of the fabrication process are illustrated in the “Materials and methods” section. It is noted that the traditional film deposition method is inappropriate for our case, because the silicon film obtained with this technique is amorphous. For the optical measurement of the sample, LCP light is generated by a quarter waveplate (QWP) and a polarizer in front of the sample. The light scattered by the metasurface is collected by a ×10/0.25 objective and filtered with another QWP and polarizer pair, which allows only the light with RCP to pass through. The details of the optical measurements are presented in the “Materials and methods” section. Figure 2c shows the scanning electron microscopy (SEM) images of the fabricated c-silicon metasurface using nanoblocks *S*_B_, *S*_G_, and *S*_R_ and their corresponding measured optical images, which can successfully produce the three primary RGB colours blue, green, and red, respectively.

### Continuous intensity tuning of each RGB colour component

With the three primary RGB colours, we then focus on the intensity (brightness) modification of each RGB component. Since the transmission magnitude of a periodical metasurface with a single nanoblock unit is unchangeable, we consider two c-silicon nanoblocks with rotation angles of *φ*_1_ and *φ*_2_ in one unit cell (Fig. 2d). We refer to this unit cell as a double-nanoblock cell (DNC) hereafter. When an LCP beam passes through a nanoblock with a rotation angle of *φ*, it will add a geometric phase of 2*φ* to the transmitted light with cross-polarisation (RCP). Τhe transmission coefficient of the cross-polarised light through the DNC is

1 $$E \propto {\mathrm{e}}^{{\mathrm{2}}{\it{i\varphi }}_1} + {\mathrm{e}}^{2i{\it{\varphi }}_2} = 2\cos \,\delta {\mathrm {e}}^{i(2{\it{\varphi }}_1 + \delta )}$$

where *δ* is the rotation angle difference between the two nanoblocks, *δ* = *φ*_2_ − *φ*_1_. The intensity of the transmitted light is proportional to cos^2^ *δ*. Therefore, by altering the rotation angle difference *δ* between the two nanoblocks, the transmission intensity can be modified. In the simulation, the period *P* is set at 550 nm so that the coupling between the two nanoblocks is negligible. The reduction of the period can increase the coupling between the two nanoblocks, which may lead to a deviation from Eq. (1). Figure 2e shows the dependence of the simulated zero-order transmission of the DNC on *δ*, which fits the theoretical prediction of cos^2^ *δ* very well. Here, we calculate only the zero-order transmission, because the higher diffraction orders cannot be collected by the objective with N.A. = 0.25. Due to the weak coupling and the magnetic resonance inside the nanoblock (Supplementary Fig. S6), the *δ* value affects only the intensity and has little influence on the resonance properties of the DNC. Therefore, the intensity can be modified while nearly maintaining the optical spectral shape. Figure 2f–h show the simulated and measured zero-order transmission spectra of the DNC under different *δ* values with nanoblocks *S*_B_ (Fig. 2f), *S*_G_ (Fig. 2g) and *S*_R_ (Fig. 2h). The magnitude of the resonance peak decreases with increasing *δ.* The discrepancy between the measured and simulated transmission spectra possibly comes from the fabrication imperfections and measurement errors. The measured optical images also demonstrate the continuous modification of the intensity with nearly unchanged hue and saturation.

In addition to the intensity, the phase can also be independently controlled^35,36^. Equation (1) indicates that the phase of the transmitted light is 2*φ*_1_ + *δ*, which can cover the whole 2*π* phase range via alteration of the rotation angle *φ*_1._ The simulated phases vs. *φ*_1_ with different *δ* values agree well with the prediction (Fig. 2i). For the curves in Fig. 2i, both parameters *φ*_1_ and *φ*_2_ are varied so that the intensity and phase terms are independently controlled. The independent control of the intensity and phase provides a route for colour printing and hologram integration in the same metasurface structure (as discussed later).

### RGB colour mixing for HSB colour tuning

**Fig. 3:**
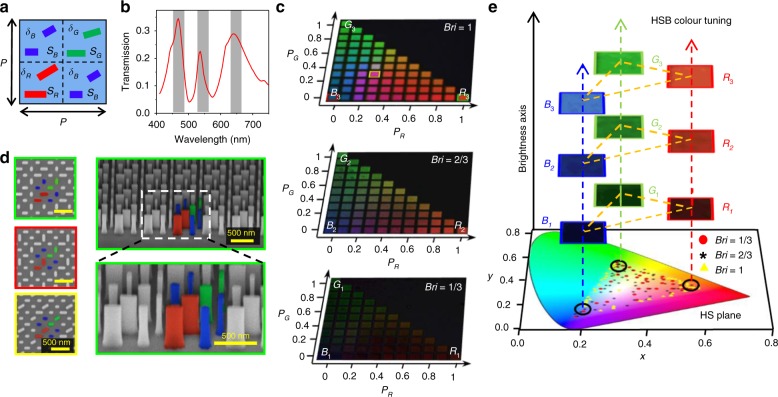
RGB colour mixing for HSB tuning. **a** Schematic of the RGB unit consisting of multiplexed c-silicon nanoblocks for HSB colour tuning. The unit consists of four DNCs, with one DNC of *S*_R_, one DNC of *S*_G_, and two DNCs of *S*_B_. The *δ* values of the two DNCs of *S*_B_ are the same. The period *P* = 700 nm. **b** The simulated zero-order transmission with cross-polarisation for the RGB unit with *δ*_B_ = 0, *δ*_G_ = 0 and *δ*_R_ = 0. **c** Colour palette obtained by stepwise tuning the proportion of red (*P*_r_), green (*P*_g_) and blue (*P*_b_) components in the RGB unit under three different brightnesses (Bri): 1, 2/3 and 1/3. The proportion of blue intensity *P*_b_ is obtained as *P*_b_ = 1 − *P*_r_ − *P*_g_. **d** Top and tilted (45°) views of SEM images of the partial colour squares indicated by the square solid lines in the top figure of **c**. The added colours indicate the blue, green and red components in the RGB unit. **e** The corresponding positions in the CIE 1931 colour space of the data from **c**. The data with Bri = 1, 2/3 and 1/3 are indicated by yellow triangles, black asterisks and red circles, respectively

HSB colour can be produced by mixing the three RGB primary colours with different proportions into one unit (Fig. 3a). The RGB unit consists of four sub-DNC units of nanoblocks *S*_B_, *S*_G_ and *S*_R_. Because the transmission of nanoblock *S*_B_ is much smaller than that of *S*_G_ and *S*_R_, two DNCs of *S*_B_ are arranged in the unit cell to enhance the transmission at the wavelength of blue. Figure 3b shows the zero-order transmission spectrum when the intensities of all the sub-DNCs in the RGB unit are chosen at the maximum value. Three transmission peaks can be observed. Simulated magnetic fields (Supplementary Fig. S7) indicate that the three peaks arise from the resonances of nanoblocks *S*_B_, *S*_G_, and *S*_R_, respectively. Moreover, the transmission at the resonance wavelength of *S*_B_ is largely enhanced due to the introduction of two DNCs of *S*_B_ in the RGB unit. The intensities of the blue, green, and red components in the RGB unit can be on-demand controlled by their orientation angle differences *δ*_B_, *δ*_G_, and *δ*_R_, respectively (Supplementary Fig. S8).

To demonstrate the capability for the HSB tuning of colours, we fabricate the c-silicon metasurfaces using the RGB unit with different *δ* values and arrange them in different palettes (Fig. 3c). In the palettes, the *x* and *y* coordinates correspond to the proportions of the red (*P*_R_) and green (*P*_G_) components in the RGB unit. The proportion of the blue component is *P*_B_ = 1 − *P*_R_ − *P*_G._ Since *P*_B_ must be positive, only half of the square palette exists. The intensities of the three RGB components are then normalised to their maximal value *P*_M_ = max(*P*_B_, *P*_R_, *P*_G_). With the introduced brightness (Bri) dimension, the *δ* values of the RGB unit are set as cos^2^ *δ*_i_ = Bri·*P*_*i*_/*P*_M_, where *i* = B, G, R. Figure 3c shows the measured optical images of the palettes with different brightnesses Bri = 1, 2/3 and 1/3 under the same illumination conditions. The brightness of the palettes clearly decreases with decreasing Bri. Figure 3d shows the SEM figures of partial colour squares indicated by the solid-line squares in Fig. 3c. The c-silicon nanoblock can be fabricated with the desired anisotropy and straight sidewall, which is important for the desired colour display. The HS values of the three palettes in Fig. 3c are displayed in the CIE 1931 colour space in Fig. 3e, all of which can cover a wide gamut around the white centre, demonstrating the capacity for full colour generation. In addition, the brightness tuning of our method extends a colour only in the HS plane to a 3D space that can cover a whole HSB colour space.

**Fig. 4:**
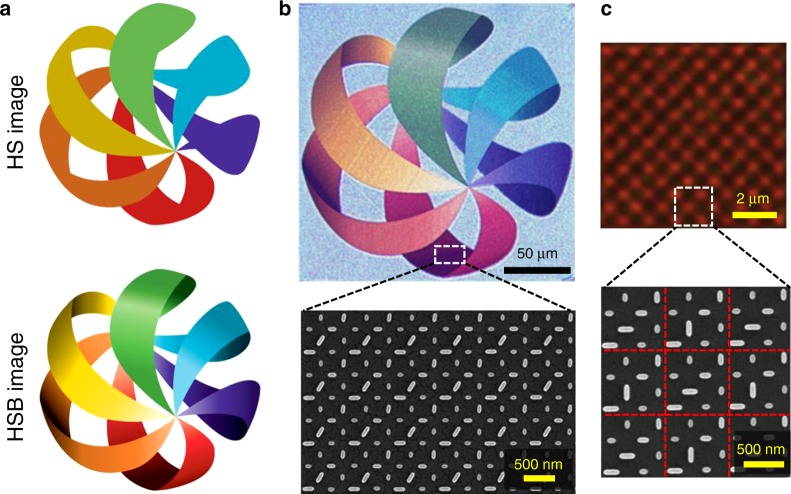
Demonstration of HSB printing image and sub-micron resolution test. **a** Comparison between HS and HSB images (logo reproduced with permission from the copyright holder Australian Trend Forecast). Due to the lack of a brightness dimension, the HS image cannot display the chiaroscuro information. **b** Optical image and SEM image of the logo based on the c-silicon metasurface. The optical image is measured with a ×10/0.25 objective. **c** Sub-micron resolution test in the form of checkboards using a ×50/0.75 objective. The dashed red lines indicate the boundaries of each RGB pixel

The generation of the HSB colour is important for image technology. From the perspective of colour imaging, the colour brightness can give the right chiaroscuro of an image, which can be the artistic essence or key merit of a picture. For example, comparing the HS and HSB modes of the same image (Fig. 4a), one can find that the right chiaroscuro allows the stereoscopic impression of the figure, making the HSB image much more vivid and artistic. Figure 4b shows the experimental demonstration of printing HSB colour images using our method. The measured optical image clearly indicates the reproduction of the HSB image with varied colours and chiaroscuro information, which is important for the display of the 3D stereoscopic effect. Furthermore, to demonstrate the colour pixel resolution at the sub-micron level, we patterned a checkerboard structure with the red (*δ*_B_ = *δ*_G_ = *π*/2, *δ*_R_ = 0) and black (*δ*_B_ = *δ*_G_ = *δ*_R_ = *π*/2) colours alternated. Each pixel in the checkerboard consists of only one RGB unit with a 700 nm length. Nevertheless, the checkerboard colour patterns can still be preserved (Fig. 4c, using a ×50/0.75 objective), demonstrating the sub-micron resolution of our metasurface image.

### Integration of HSB colour printing and full-colour hologram

**Fig. 5:**
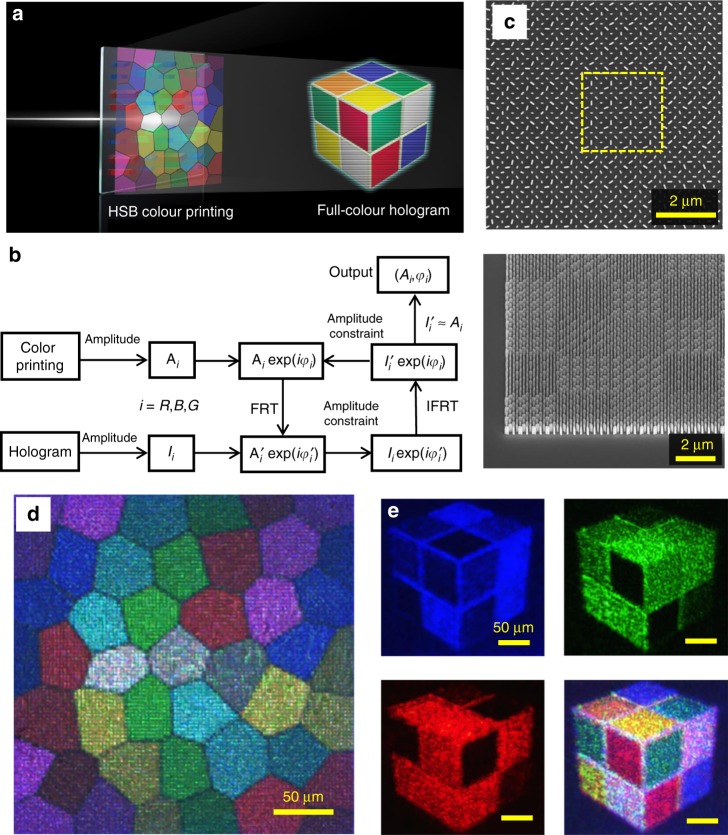
Integration of HSB colour printing and full-colour hologram. **a** Schematic of the metasurface for the integration of HSB colour printing and the full-colour hologram image. The hologram plane is designed at 1075 μm above the colour printing image. **b** Flow chart of the metasurface design for the integration of colour printing and holography. *A*_*i*_ and *I*_*i*_ are the intensities of the three RGB components (*i* = R, G, B) in the colour printing image and hologram, respectively. FRT and IFRT represent the Fresnel transformation and inverse Fresnel transformation, respectively. **c** Top (upper) and tilted (45°, lower) views of the fabricated metasurface. The dashed yellow line indicates an enlarged pixel, which consists of 4 × 4 RGB units. **d** Measured optical printing images. **e** Measured hologram images illuminated by lasers with a wavelength at only 473 nm (top left), 532 nm (top right), 633 nm (bottom left) and all three simultaneously (bottom right)

We further show how to integrate arbitrary HSB colour printing and a full-colour hologram image in a single metasurface (Fig. 5a). This integration is enabled by the independent control of the intensity and phase of the three primary RGB colours in our metasurface. It is worth mentioning that recently, a two-layer structure with a dielectric phase plate and a dielectric pillar array for structural colour elements was proposed for the integration of colour printing and holography^13^. However, in their designs, the structural colours were altered by varying the dielectric pillar array dimensions, such as the height, diameter, and pitch, lacking the ability of brightness tuning, which led to the formation of HS images only. In addition, since their structural colours were formed by the transmissive-guided mode of the colour filter array, it natively exhibited a large linewidth of over 150 nm. Many attempts to search for a design are needed to carry out colour printing and holography simultaneously. Therefore, the number of choices of this design is very limited, prohibiting the integration of arbitrary colour printing and full-colour hologram images. These limits will greatly hinder future application of this integration strategy.

Figure 5b shows a flow chart of our arbitrary HSB colour printing and full-colour hologram integration process using a modified Gerchberg–Saxton algorithm^37^. First, the intensities of the three RGB components of the colour print image *A*_*i*_ (*i* = R, G, B) and hologram *I*_*i*_ (*i* = R, G, B) are extracted. Then, a random phase *φ*_i_ is added to the intensities of each component of colour printing *A*_*i*_. The optical field is then propagated to the hologram plane using a Fresnel transform (FRT). The intensity distribution $A_i^\prime$ is then substituted with the desired hologram intensity *I*_*i*_ and propagated backward to the printing image plane with the inversed Fresnel transform (IFRT). At the printing image plane, the intensity constraint is also taken. After several iterations, if the deviation between the obtained intensity $I_i^\prime$ and the target *A*_*i*_ is sufficiently small, the phase distribution *φ*_*i*_ at the printing image plane is returned. We choose a figure of stained glass with various colours and intensities (i.e., HSB figure) as the colour printing image and a pocket cube as the hologram image (Supplementary Fig. S9). The hologram plane is designed to be 1075 μm above the metasurface plane. Supplementary Fig. S10 shows the intensity and calculated phase distributions of the three RGB components at the metasurface plane. Based on the intensity *A*_*i*_ and phase *φ*_*i*_, the rotation angles of nanoblocks in the RGB unit can be obtained based on Eq. (1).

In the demonstration of HSB colour printing in Fig. 4, we set *φ*_1_ = 0 for all RGB units so that the phase 2*φ*_1_ + *δ* of each RGB pixel is determined only by *δ.* Since the intensity (cos^2^ *δ*) of the colour printing image usually has a slow and continuous spatial variation, the phase difference between adjacent pixels is negligible. However, when the hologram is integrated with the colour printing image, the phase difference between adjacent pixels becomes significant. In the experiment, we use an objective with an N.A. of 0.25, which has a spatial resolution of ~1–1.5 μm. Therefore, the measured image does not correspond one-to-one to the original intensity of each RGB unit (0.7 μm × 0.7 μm) but includes the interference of several adjacent RGB units. If the phase difference between adjacent pixels changes slowly, for example, the case in Fig. 4, the measured and the designed input images can be almost identical. Otherwise, the measured image will differ significantly from the designed colour printing image.

To eliminate the effect of pixel interference on the final colour printing image, an enlarged pixel with 4 × 4 RGB units is created, in which all the RGB units are the same. The enlarged pixel has dimensions of 2.8 μm × 2.8 μm, as shown by the yellow dashed lines in Fig. 5c. This pixel can be seen more clearly from a tilted view (bottom panel of Fig. 5c). The final fabricated sample consists of 96 × 96 enlarged pixels, with a total size of 268.8 μm × 268.8 μm. Figure 5d shows the measured colour printing image, where the different colours and brightness of the stained glass are well reproduced. Because of the phase differences between adjacent enlarged pixels, the interference between them causes the printing image to be pixelated. Increasing the dimensions of the enlarged pixel may reduce the visually pixelated effect but can result in a reduced viewing angle in holography. For holography, three free-space lasers with wavelengths at 473, 532, and 633 nm are used. The focal plane of the objective is tuned to be 1075 μm above the metasurface plane. When only one of the three lasers is used, the corresponding RGB component of the designed hologram can be obtained (Fig. 5e). If all three lasers simultaneously illuminate the metasurface, the colour mixing of the three RGB holograms can generate a colourful pocket cube with six kinds of colours (bottom-right panel in Fig. 5e). Because of the existence of the laser speckles in the holographic image, these colours are not as pure and spatially homogeneous as we have designed. The measured efficiencies of the hologram image are 6.4% at 473 nm, 7.8% at 532 nm, and 10.3% at 633 nm, which are much lower than the simulated efficiencies shown in Fig. 3b. The reason is that the optical efficiency of the hologram is also influenced by the transmission amplitude through the metasurface, which is determined by the brightness of the colour printing image. Since the input colour printing image has a varied brightness distribution, the transmission amplitude through the metasurface cannot reach the maximal values at all positions, therefore limiting the optical efficiency of the hologram. If one is interested only in the hologram functionality, the theoretical limits of the optical efficiencies at the red, green, and blue wavelengths are 28%, 22%, and 35%, respectively, according to Fig. 3b.

## Discussion

The main advances are discussed here. First, we have demonstrated the realisation of a real HSB colour printing technique, which may bring about revolutionary effects in the field of colour printing. For the previous printing image approaches based on nanostructure colours, although numerous advantages have been demonstrated, they cannot cause all of the real colours to reappear in the practical world. Additionally, due to the lack of brightness control, previous approaches can hardly exhibit the right chiaroscuro of an image, which means that they cannot make a two-dimensional printing image look truly three-dimensional and fail to reproduce the pictures with the chiaroscuro, such as the masterpieces of Leonardo Davinci and Caravaggio, or any photos of the real world. However, our approach brings about a solution to eliminate these defects of structural colours, greatly expanding its application range. Second, HSB colour printing can be achieved based on sub-micron pixels, indicating high resolution for image generation and high capacity for optical information storage. Third, we further demonstrate the integration of HSB colour printing and full-colour hologram images in a metasurface chip. Compared to previous integration work on colour printing and holography, which suffers from limited colour options^13^, our work can achieve arbitrary colours in both HSB colour printing and holography. Moreover, our results unlock the third dimension of brightness. These advancements make our approach significantly robust and versatile. For example, if the printing image serves as the cover image and the real information is recorded by the hologram image, a completely different nano-steganography is introduced. We believe that our work has potential applications in a wide range of fields, such as image display techniques, multi-functional holograms and complex electromagnetic field generation.

## Materials and methods

### Numerical simulation

The numerical simulation is calculated by commercial finite-difference time-domain (FDTD) software (FDTD solutions, Lumerical Inc.). The periodic boundary condition and perfectly matched layer are employed for the horizontal (*x*- and *y*-) and vertical (*z*-) directions in the simulation, respectively. The refractive index of SiO_2_ used is 1.45. The dielectric constants of c-silicon and a-silicon are taken from Palik^38^ and Pierce^39^, respectively. NOA61 is neglected in the simulation because the refractive index of NOA61 (~1.55) is close to that of SiO_2_. To obtain the transmission magnitude with cross-polarisation, the *E*_*x*_ and *E*_*y*_ components of the zero-order-transmitted diffraction are calculated. The cross-polarisation (RCP) is then written as $\sqrt 2 (E_x + 1iE_y)/2$.

### Transfer and fabrication of c-silicon metasurface

To transfer the c-silicon onto the SiO_2_ substrate, we first deposit 800 nm-thick SiO_2_ using inductively coupled plasma chemical vapour deposition (ICP-CVD) on an SOI wafer containing a 1250 nm c-silicon layer on 300 nm of SiO_2_. Then, the sample is spin-coated with adhesive NOA61, followed by bonding to the fused SiO_2_ substrate. Next, the sample is exposed to UV light to cross-link the NOA61 polymer for 4 h, followed by baking for 3 days at 50 °C. The silicon substrate is then polished down to 40 μm and completely removed by DRIE. The box SiO_2_ layer is removed with HF acid. Then, ICP is used to reduce the thickness of the c-silicon layer to 600 nm.

The fabrication of the c-silicon metasurface is carried out with EBL. A 320 nm-thick HSQ layer is first spin-coated at 4000 rpm onto the c-silicon and then baked on a hot plate for 5 min at 90 °C. Then, a 30 nm-thick aluminium layer (thermal evaporation) is deposited to serve as the charge dissipation layer. Next, the pattern is exposed using a Raith Vistec EBPG-5000plusES electron beam writer at 100 keV. After exposure, the aluminium layer is removed by 5% phosphoric acid, and the resist is developed with tetramethylammonium hydroxide. Finally, the sample is etched using ICP. A schematic of the transfer and fabrication process of the c-silicon metasurface is shown in Supplementary Fig. S11.

### Optical measurement

For the optical measurement of the sample, an LCP beam is generated by a QWP and a polarizer in front of the sample. The light scattered by the metasurface is collected by a ×10/0.25 objective and filtered with another QWP and polarizer pair, which allows only the light with RCP to pass through. A white source (MNWHL4, Thorlabs Inc., optical spectrum shown in Supplementary Fig. S12) is used to measure the colour printing image, and three free-space lasers at wavelengths of 473, 532 and 633 nm are used for the hologram measurement. By controlling the position of the objective with the sample, the images at the metasurface plane and hologram plane can be obtained by a CMOS colour camera. To measure the transmission spectra, the CMOS is replaced with a spectrometer and a charge-coupled device (CCD). The optical setup is shown in Supplementary Fig. S13.

## Supplementary information

SUPPLEMENTARY INFORMATION for Full-colour nanoprint-hologram synchronous metasurface with arbitrary hue-saturation-brightness control
