# Supplementary material for: Full-colour nanoprint-hologram synchronous metasurface with arbitrary hue-saturation-brightness control
Source: Light Sci Appl. 2019 Oct 23;8:95. doi: 10.1038/s41377-019-0206-2 (PMC6813292; doi:10.1038/s41377-019-0206-2)
Supplement: Supplementary file 1 — SUPPLEMENTARY INFORMATION for Full-colour nanoprint-hologram synchronous metasurface with arbitrary hue-saturation-brightness control [file 41377_2019_206_MOESM1_ESM.docx]

Supplementary Information For

**Full-colour nanoprint-hologram synchronous metasurface with arbitrary hue-saturation-brightness control**

Yanjun Bao^1^, Ying Yu^1^, Haofei Xu^1^, Chao Guo^1^, Juntao Li^1^, Shang Sun^2^, Zhang-Kai Zhou^1,*^, Cheng-Wei Qiu^2, 3,*^ and Xue-Hua Wang^1,*^

^1^State Key Laboratory of Optoelectronic Materials and Technologies, School of Physics, Sun Yat-sen University, Guangzhou 510275, China

^2^Department of Electrical and Computer Engineering, National University of Singapore, 4 Engineering Drive 3, Singapore 117583

^3^NUS Suzhou Research Institute (NUSRI), Suzhou Industrial Park, Suzhou 215123, China

*Email: zhouzhk@mail.sysu.edu.cn; chengwei.qiu@nus.edu.sg; wangxueh@mail.sysu.edu.cn


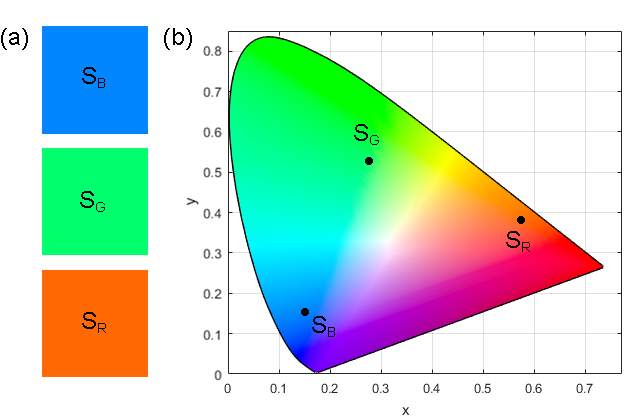


**Supplementary Fig. S1** (a) The calculated colors of *S*_B_, *S*_G_ and *S*_R_ based on the transmission spectra in Figure 2b. (b) The Corresponding positions in the CIE 1931 color space of colors in (a).


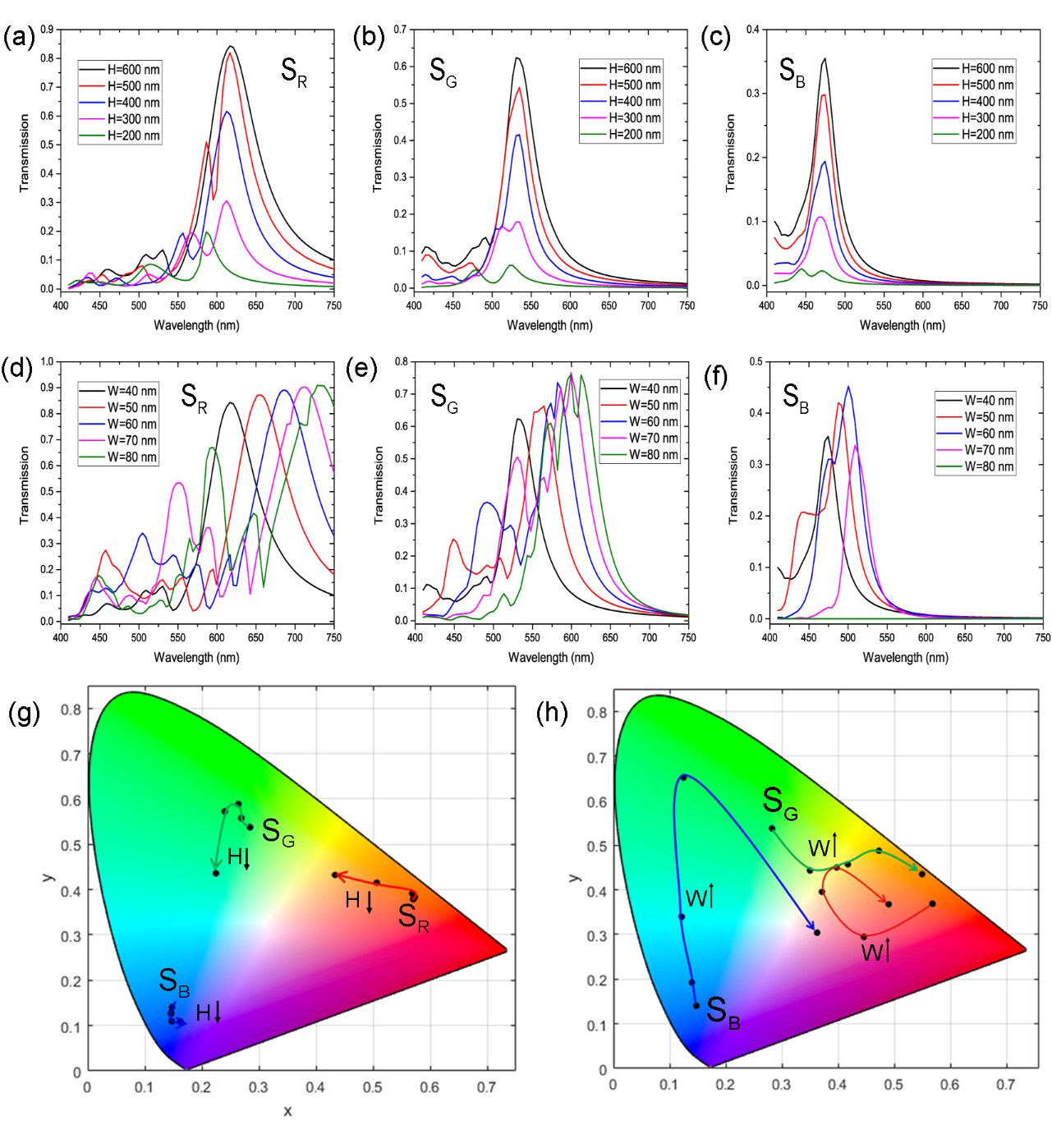


**Supplementary Fig. S2** (a-c) The calculated transmission spectra of nanoblocks *S*_B_, *S*_G_ and *S*_R_ for different height *H*. With the decreasing of the *H*, the transmission peaks decrease and become broadband. (d-f) The calculated transmission spectra of nanoblocks *S*_B_, *S*_G_ and *S*_R_ for different width *W*. With the increasing of the *W*, the transmission peaks become broadband and split. (g-h) The evolution of the positions in the CIE 1931 color space of nanoblocks *S*_B_, *S*_G_ and *S*_R_ when decreasing *H* (g) and increasing *W* (h) based on the transmission spectra in (a-c) and (d-f), respectively. The arrows indicate the evolution trends.


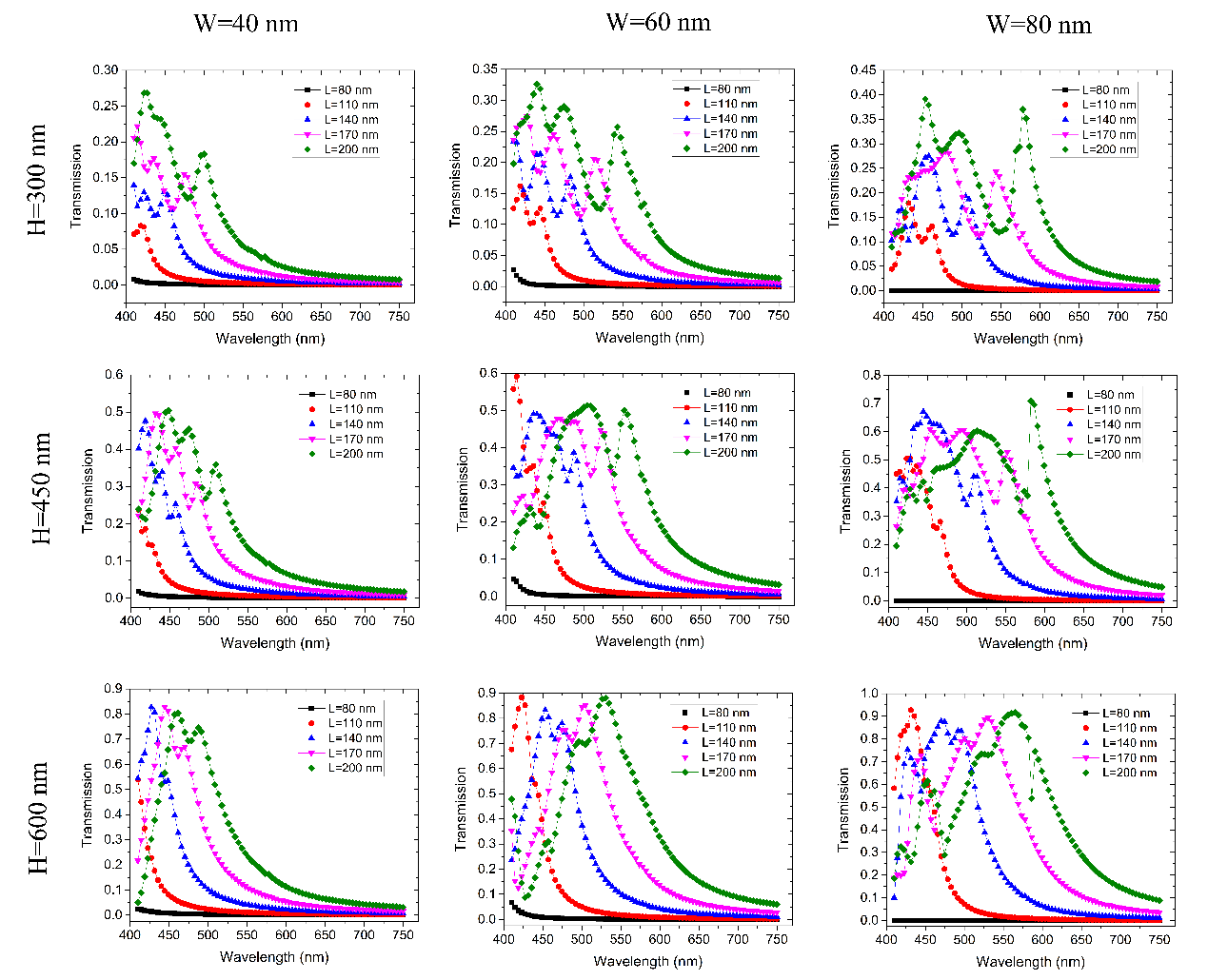


**Supplementary Fig. S3** The calculated cross-polarized transmission spectra of a periodical structure (400 nm period) using TiO_2_ nanoblock by sweeping the height H, width W and length L. The simulation shows that there is no single narrow prominent resonance presented in the spectra.


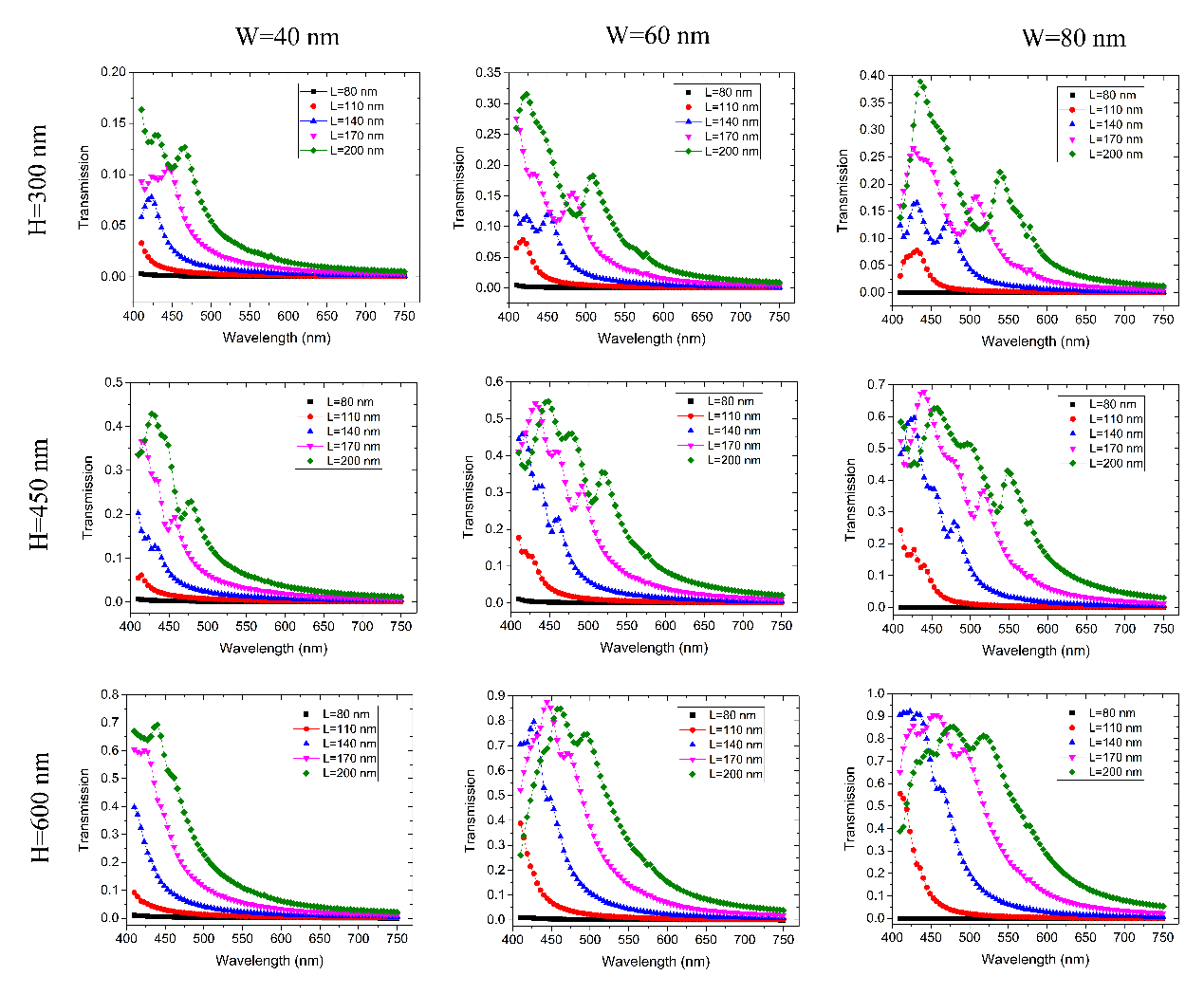


**Supplementary Fig. S4** The calculated cross-polarized transmission spectra of a periodical structure (400 nm period) using GaN nanoblock by sweeping the height H, width W and length L. The simulation shows that there is no single narrow prominent resonance presented in the spectra.


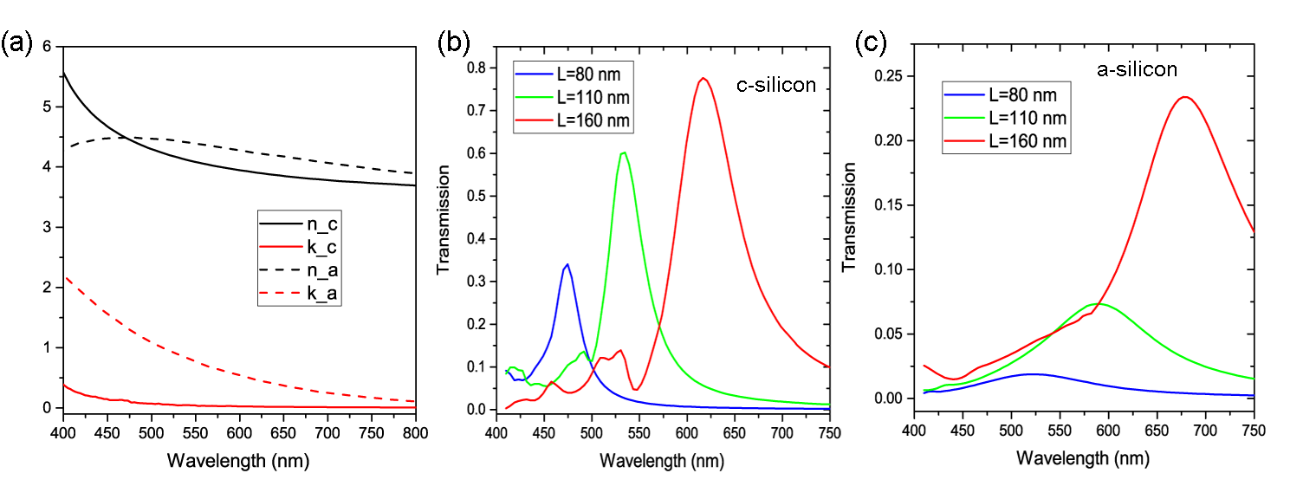


**Supplementary Fig. S5** (a) The real (black) and imaginary (red) part of the refractive index of crystal-silicon (c-silicon, solid lines) and amorphous silicon (a-silicon, dashed lines). The refractive index of c-silicon and a-silicon are taken from Palik and Pierce, respectively. (b-c) The calculated transmission spectra of nanoblocks with *L*=80 nm, *L*=110 nm and *L*=160 nm for c-silicon (b) and a-silicon (c).


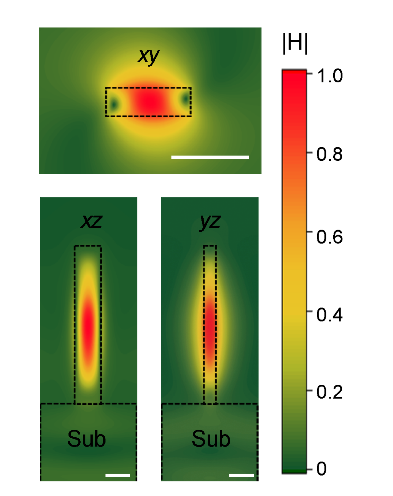


**Supplementary Fig. S6** The simulated magnetic field distributions in *xy*, *xz* and *yz* planes at the resonance wavelength of nanoblock S_G_. The dashed black lines indicate the domains of silicon nanoblock and substrate. Scale bars: 100 nm. The magnetic field is mainly concentrated inside of the silicon nanoblock, which indicates that the resonance peak is arisen from the magnetic resonance of the silicon nanoblock.


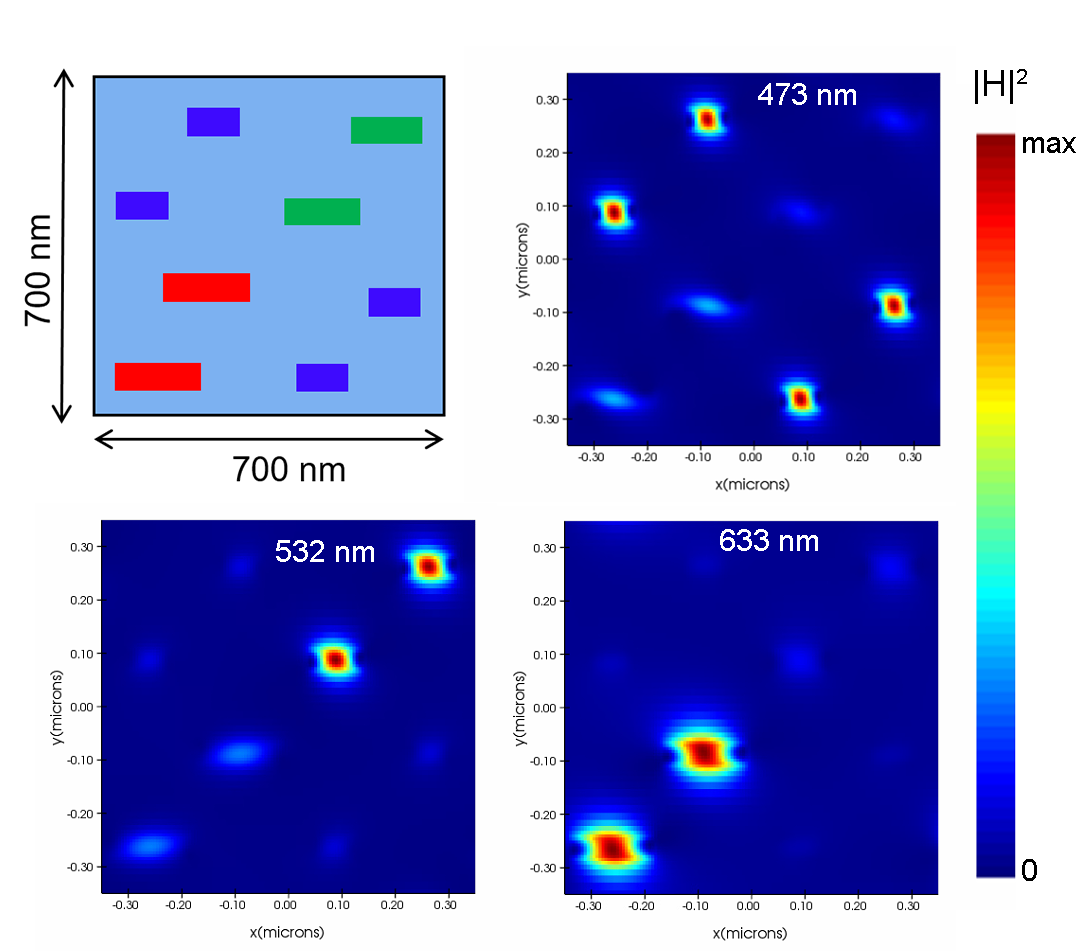


**Supplementary Fig. S7** The simulated magnetic field at 473 nm, 532 nm and 633 nm for the RGB unit when the intensities of all the sub-DNCs in the RGB unit are chosen at the maximum value (*δ*_B_=0, *δ*_G_=0 and *δ*_R_=0).


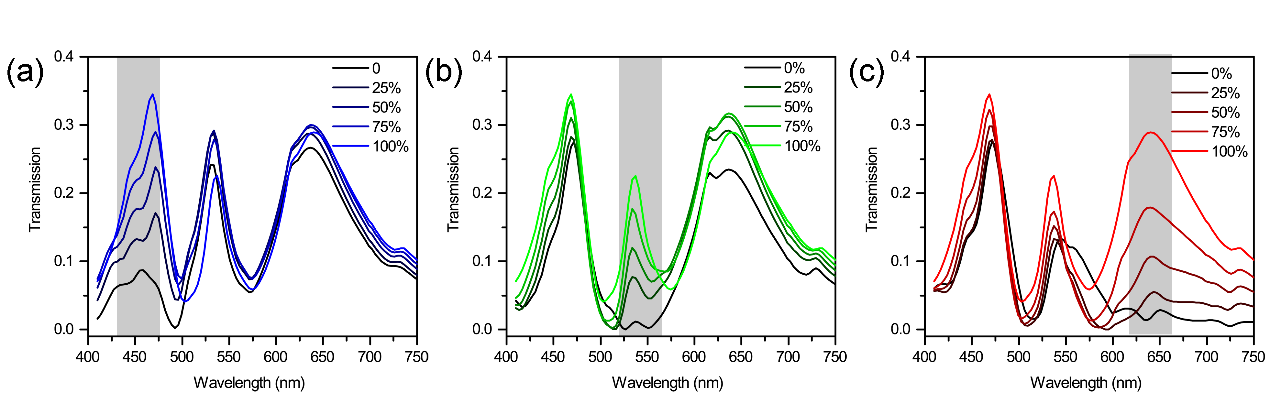


**Supplementary Fig. S8** (a-c) The calculated zero-order transmission spectra of RGB unit metasurface when the intensity of blue (a), green (b) and red (c) component changes from zero to maximum, respectively. For each sub-figure, when the intensity of one component changes, the intensities of the other two are maintained at the maximum.


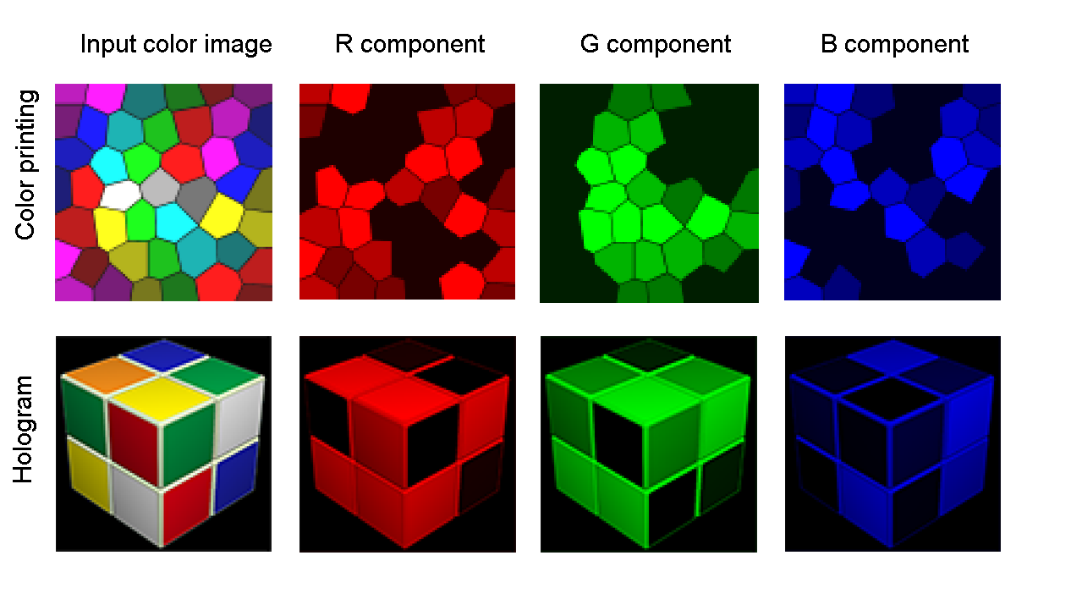


**Supplementary Fig. S9** The origin images and the red, green and blue components of the input color printing and hologram.


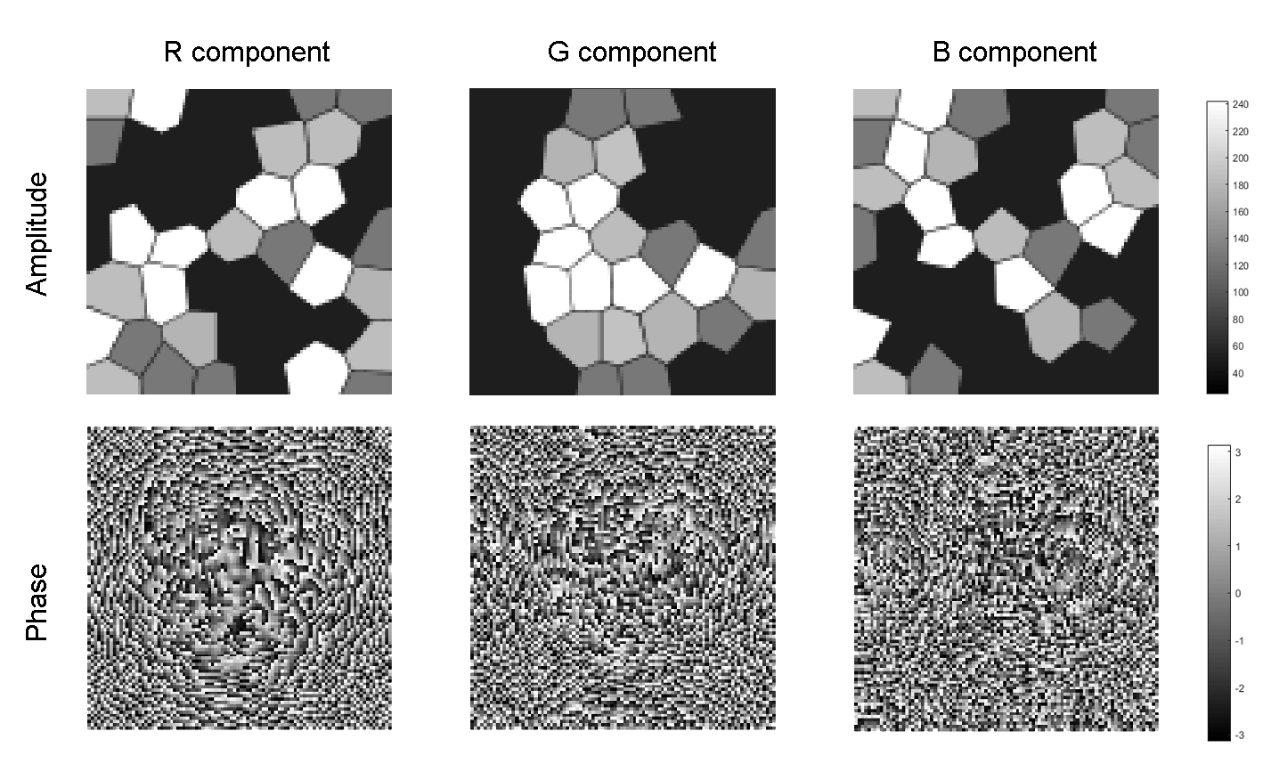


**Supplementary Fig. S10** The amplitude and the calculated phase distributions of the metasurface for the red, green and blue component of the RGB unit. The pixel number of the input images is resized to 96×96.


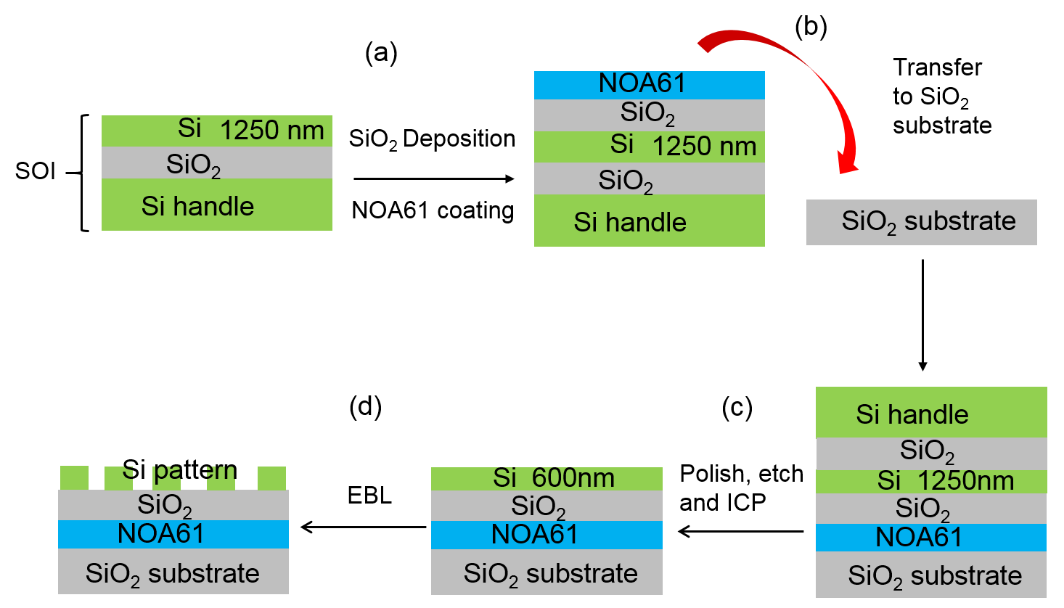


**Supplementary Fig. S11** Schematic illustration of the c-silicon transfer process and sample fabrication. (a) Deposition of silica on an SOI wafer using inductively coupled plasma chemical vapor deposition (ICP-CVD) and spin-coating of the adhesive NOA61. The thickness of the top Si of SOI is 1250 nm. (b) The bonding SOI with fused SiO_2_ substrate. Then the sample is exposed with UV light for 4 h, followed by baking for 3 days at 50 °C. (c) Polishing the silicon substrate to ∼40 μm, removing the remaining silicon substrate by deep reactive ion etching (DRIE) and the box SiO_2_ layer with HF acid. Then ICP is used to reduce the thickness of c-silicon layer to 600 nm. (d) The EBL process is used to obtain the final patterns.


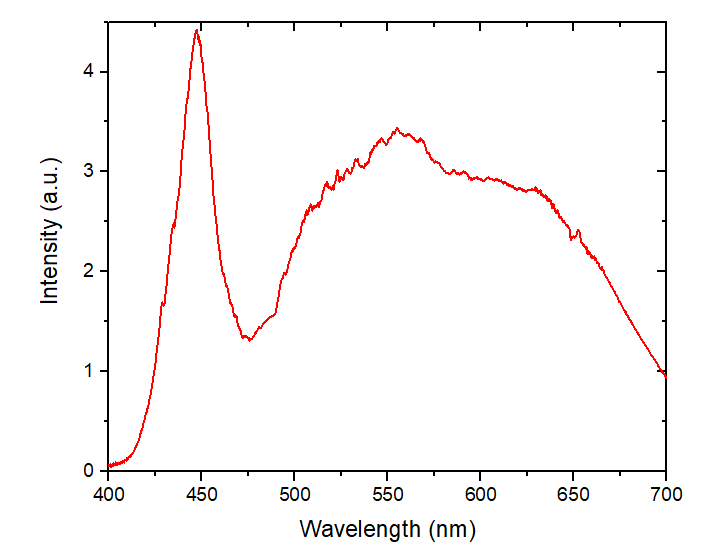


**Supplementary Fig. S12** Spectrum of the white source used in the experiment.


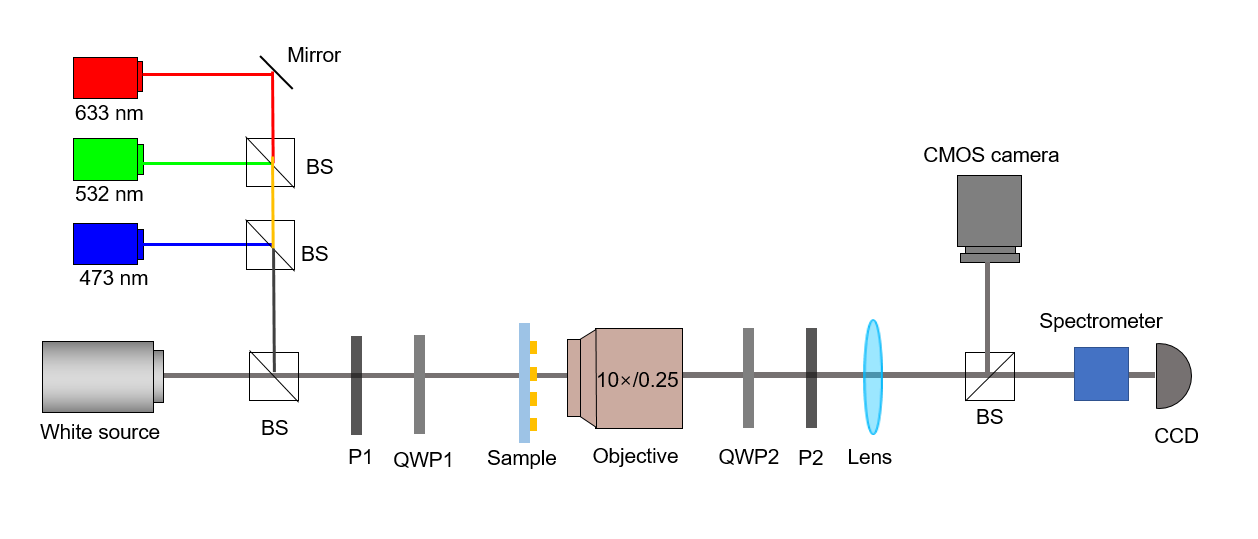


**Supplementary Fig. S13** Experimental optical setup for measuring the optical images of printing image (hologram) and the transmission spectrum. The system includes a white source, three laser at wavelengths 473, 532, 633 nm, two polarizers, two quarter waveplates (QWP), several lens, an objective, a CMOS camera, a spectrometer and a CCD. The transmitted light is collected by 10×/0.25 objective and coupled into the CMOS camera for optical imaging or the spectrometer (CCD) for transmission spectrum measurement.
